# Prevalence, Associated Factors, and Epidemiological Profile of Anemia Among Adults in a University Referral Hospital, Eastern Morocco

**DOI:** 10.3390/epidemiologia7020033

**Published:** 2026-03-02

**Authors:** Nawal Ouahbi, Khalid Serraj Andaloussi, Habiba Benneser Alaoui

**Affiliations:** Laboratory of Immunohematology and Cellular Therapy, Faculty of Medicine and Pharmacy, Mohammed I University, Oujda 60049, Morocco; serrajkhalid@yahoo.fr (K.S.A.); habiba160@hotmail.com (H.B.A.)

**Keywords:** anemia, prevalence, hospitalized adult patients, associated factors, eastern Morocco

## Abstract

Background/Objectives: Anemia is a significant global public health problem that may signal a serious underlying health condition. However, its epidemiological profile among Moroccan adults of both sexes remains poorly documented. This study aims to determine the prevalence and associated factors, and to identify the profiles of observed anemia among hospitalized patients at the University Hospital Mohammed VI of Oujda. Methods: A prospective cross-sectional study was conducted among adult inpatients (≥18 years) admitted between February 2024 and April 2025. Sociodemographic, lifestyle, and clinical data were collected using structured questionnaires and hospital records. Statistical analyses were performed using SPSS version 21, applying the Mann–Whitney U test for quantitative variables and the Chi-square or Fisher’s exact test for categorical variables. Results: A total of 446 adult patients were included in the study. The overall prevalence of anemia was 30.3%, affecting 31.9% of men and 28.4% of women. The highest prevalence (45.3%) was observed among participants aged 50 years and older. The services with the highest rates were the thoracic surgery, pneumology, and burns and reconstructive surgery departments. Significant associations with anemia were identified for age group (*p* = 0.004), hospital department (*p* = 0.028), presence of medical comorbidities (*p* < 0.001), and type of diagnosis at admission (*p* = 0.019). The predominant forms of anemia were mild anemia (62.2%), and normocytic normochromic anemia was the most frequent morphological type (55.6%). Conclusions: Anemia is a frequent comorbidity among hospitalized adults. Systematic screening upon admission and appropriate management during hospitalization are essential to reduce anemia-related complications and improve patient outcomes.

## 1. Introduction

Anemia is a major global health concern, affecting around two billion people, representing nearly a quarter of the world’s population [1]. It ranks as the third leading cause of disability worldwide [1,2] and is among the ten most serious health problems, particularly in low- and middle-income countries [3,4]. The World Health Organization (WHO) estimates that anemia causes roughly one million deaths each year, with the majority of cases occurring in Africa and Southeast Asia [5].

The etiology of anemia is multifactorial and context-dependent. Globally, iron deficiency remains the leading cause, although deficiencies in vitamin B12 and folate are also important contributors [4,6,7]. Other causes include hemoglobinopathies, hemolytic anemias, anemia of inflammation, chronic diseases, and parasitic infections, with their distribution varying by age, sex, and socioeconomic status [8,9,10]. In hospital settings, additional factors such as acute or chronic blood loss, comorbidities (e.g., renal insufficiency, heart failure), and nutritional deficiencies further increase anemia risk [11,12,13].

Clinically, anemia is often asymptomatic and detected incidentally through routine complete blood count. When present, symptoms may include fatigue, dizziness, dyspnea, headaches, and cognitive impairment, though these are nonspecific [14]. Diagnosis relies on hemoglobin measurement, with WHO defining anemia in adults as hemoglobin <13 g/dL for men and <12 g/dL for non-pregnant women [2].

Anemia can be classified both pathophysiologically, reflecting decreased red cell production, increased destruction, or blood loss [15], and morphologically, as microcytic, normocytic, or macrocytic anemia [16]. Accurate classification is essential to guide appropriate management and treatment.

In hospitalized patients, anemia is highly prevalent, ranging from 13% [17] to 71% [13]. Its clinical impact is substantial, as it consistently correlates with increased morbidity, mortality, postoperative complications, prolonged length of stay, and higher healthcare costs [18,19,20,21]. Preoperative anemia independently increases the risk of infections, acute kidney injury, and postoperative mortality [18,19,20,22], while persistent anemia at discharge predicts early readmission [22]. In non-surgical populations, anemia has a similarly significant effect, particularly in patients hospitalized for congestive heart failure, where it stands out as an independent predictor of mortality [23]. Overall, the evidence supports routine screening and timely management of anemia in hospitalized patients [21].

In Morocco, anemia is prevalent and etiologically diverse. A cross-sectional study in the M’diq–Fnideq–Martil prefecture reported anemia prevalence of 52.4% in adults [24]. Hospital-based data from Meknès indicate that iron deficiency accounts for approximately 60% of cases, followed by megaloblastic (21.33%) and hemolytic (7.33%) anemia [25]. Additionally, a retrospective study at Mohammed V Military Training Hospital in Rabat described 34 cases of pernicious anemia, demonstrating that vitamin B12 deficiency also contributes to the burden [26]. Collectively, these findings highlight the need for local epidemiological research to inform context-specific anemia management strategies.

Given its high prevalence and modifiable nature, anemia represents an important target for clinical and public health interventions. Strategies including nutritional supplementation, treatment of underlying conditions, and patient blood management can improve outcomes and reduce healthcare costs [12,18,21]. Systematic assessment of anemia prevalence and etiology is therefore critical to optimize care, particularly in Moroccan hospital settings.

This study aims to determine the prevalence, morphological patterns, and associated factors of anemia among adult inpatients at the Mohammed VI University Hospital Center in Oujda, Morocco, and to provide evidence to guide clinical practice and public health interventions.

## 2. Materials and Methods

### 2.1. Study and Population Design

A prospective descriptive and analytical cross-sectional study was conducted at Mohammed VI University Hospital in Oujda between February 2024 and April 2025. The following departments were included: ophthalmology, otolaryngology, neurosurgery, burn and reconstructive surgery, thoracic surgery, neurology, pulmonology, dermatology, and cardiology. These departments were selected because they do not primarily treat anemia and do not routinely admit patients for conditions in which anemia is a common complication. The study population consisted of adult patients (≥18 years) residing in eastern Morocco and hospitalized in one of the selected units during the study period. To be eligible, patients had to have undergone a complete blood count (CBC) upon admission. Exclusion criteria were pregnancy or postpartum, age < 18 years, psychiatric or cognitive disorders, history of blood transfusion in the previous three months, inability to communicate or respond to the questionnaire, and a complete blood count performed outside the hospital laboratory. Hospital departments with a high expected prevalence of anemia, including internal medicine, hematology, nephrology, gastroenterology, intensive care unit, and other services, were excluded a priori to minimize selection bias and to focus on services where anemia is less expected and often underdiagnosed. A total of 446 patients meeting these criteria were included.

### 2.2. Data Collection

Two data collection tools were used in this study. The first was a structured questionnaire administered face-to-face by an interviewer and previously validated through a pre-test. It collected detailed information on sociodemographic and economic characteristics (age, gender, marital status, city and place of residence, education level, occupation, type of housing, and health insurance coverage), health-related behaviors (physical activity, smoking, and alcohol consumption), and relevant medical history. The second source of data was laboratory results. More precisely, that of the CBC carried out at the time of admission to the hospital.

### 2.3. Measurements

The dependent variable was the presence of anemia among admitted patients, defined according to WHO thresholds as a hemoglobin concentration <12.0 g/dL in women and <13.0 g/dL in men [27]. For statistical analysis, this variable was recoded as binary (0 = non-anemic; 1 = anemic).

Anemia severity and morphological classifications were also based on WHO standards. Severity was categorized as mild (Hb 11–12.9 g/dL for men and 11–11.9 g/dL for women), moderate (Hb 8–10.9 g/dL for both sexes), or severe (Hb < 8 g/dL) [28]. Morphology was defined using Mean Corpuscular Volume (MCV) and Mean Corpuscular Hemoglobin Concentration (MCHC): anemia was classified as microcytic (MCV < 80 fL), normocytic (80–100 fL), or macrocytic (MCV > 100 fL), and as normochromic (MCHC 28–36 g/dL) or hypochromic (MCHC < 28 g/dL) [29].

The independent variables included in the analysis were identified based on previous literature, including demographic characteristics such as sex, age, marital status (single, married, divorced, widowed), City of residence (Oujda, Berkane, Nador, Jerrada, Taourirt, Driouch, Guercif, Figuig), Place of residence (urban, rural), and education level (illiterate, no formal (masjid), primary, secondary, higher or above). Socioeconomic data includes occupation and Health insurance coverage. Occupation was classified into the following categories: formal, informal, unemployed, and inactive. Health insurance coverage was classified according to the Moroccan social protection system, including AMO TADAMON (Social Solidarity Program), CNOPS (National Fund for Social Welfare Organizations), CNSS (National Social Security Fund), other insurance schemes, and uninsured participants.

Lifestyle characteristics were categorized as smoking status (current smoker, former smoker, non-smoker), physical activity (yes/no), duration of sport/week (less than 2 h, more than 2 h), and alcohol consumption (yes/no).

Regarding clinical and hospital data, participants’ medical history indicates the presence of one or more chronic diseases already diagnosed in patients admitted to the hospital. Related to the clinical hospital units, they were categorized as medical, surgical, or medical-surgical. The clinical classification of hospitalization diagnoses was established based on the initial diagnosis recorded at patient admission, as documented in the medical record. Diagnoses were subsequently grouped into six categories: cardio-respiratory, neoplastic, surgical, inflammatory, infectious, and other conditions. The latter category included diagnoses not fitting into the previous groups, as well as syndromes or symptoms recorded as admission diagnoses and degenerative diseases.

### 2.4. Data Analysis

Statistical analyses were performed using Microsoft Excel (2016) and the Statistical Package for the Social Sciences (version 21). Descriptive analysis was used to summarize data in tables and graphs. For qualitative variables, data were presented in frequencies. For quantitative variables, the normality of variable distribution was analyzed using the Kolmogorov–Smirnov and Shapiro–Wilk tests. Given the non-normality of hemoglobin, nonparametric tests, such as the Mann–Whitney U test, were used to compare group differences. Categorical variables were compared using the Pearson Chi-square test or the Fisher’s exact test, as appropriate. To account for multiple comparisons, *p*-values were adjusted using the Benjamini–Hochberg procedure, and a *p*-value < 0.05 was considered statistically significant.

### 2.5. Ethical Consideration

This study was conducted in accordance with the ethical principles of the 2013 Declaration of Helsinki. The research protocol was approved by the Biomedical Research Ethics Committee of the Faculty of Medicine and Pharmacy of Oujda (Reference: 55/2023. 15 January 2024). Authorization to conduct the study was also obtained from the University Hospital Mohammed VI of Oujda, and the heads of the participating hospital units were informed about the study’s objectives and procedures.

Written informed consent was obtained from all participants prior to data collection. They were informed that participation was voluntary and that all collected data would remain confidential and anonymous.

## 3. Results

### 3.1. Socio-Demographic, Economic, and Lifestyle Characteristics of Study Participants

The descriptive results of the study are presented in Table 1. A total of 446 adult patients aged 18 and above were included in the study, comprising 211 (47.3%) men and 235 (52.7%) women. The median age was 57 (IQR: 41.75–68) for all participants, with a predominance of the 60-year age group (45.3%). It was 59 (IQR: 42–69) years old for males and 54 (IQR: 42.5–69) years old for females. Participants represented all provinces of the eastern region, with Oujda accounting for the most significant proportion (43.7%) of the study population. The majority of participants originated from urban areas (68%), and were unemployed at the time of the study (71.5%). Almost half of the study participants were illiterate (49.8%), and the majority were unemployed (70.2%). Medical coverage showed that more than half of respondents were covered by the AMO Tadamon scheme (52.24%); however, a considerable proportion (19.1%) had no health insurance coverage.

Regarding lifestyle characteristics, among 446 participants, only a minority (16.8%) practiced physical activity, while the majority (83.2%) did not. Most participants were non-smokers (65%) and former smokers (27.6%). Nearly all of the participants were non-alcoholic (96.2%).

### 3.2. Epidemiological Profile of Anemia

#### 3.2.1. Prevalence of Anemia and Factors Associated

The study results show that the overall prevalence of anemia was 30.3% (135 patients), with a slight difference between men (31.9%) and women (28.4%); this difference was not statistically significant (*p*-value 0.425). Anemia was most frequent in the 60 years and above age group. This difference between age groups was statistically significant (*p* = 0.004).

Analysis of anemia status in relation to sex and age shows that the most anemic age groups for women were patients aged 60 and over, and young people aged 18–29. For men, the 60 years and above and 45–59 age groups were the most affected. Table 2 shows an increasing trend in anemia prevalence with age beginning at 45 years for both sexes, with patients aged 60 years and above showing the highest rates. This association between age and sex was statistically significant in men (*p* = 0.007) but not in women (*p* = 0.331).

The prevalence of anemia was higher in patients who did not engage in physical activity (31.5%) than in those who did (24%), and in participants who were alcohol consumers (41.2%) than in non-alcohol consumers (29.8%), as well as higher in patients with no medical coverage (34.1%) than in those with coverage. This difference in prevalence between these groups was not significant. The study of associations between anemia and other socio-demographic characteristics and lifestyle factors showed no significant associations (Table 1).

#### 3.2.2. Clinical and Hospital-Related Factors

In reference to Table 3, the distribution of study participants by hospital unit demonstrates the representativeness of all study sites. Similarly, the units with the highest representation were cardiology (16.4%), neurosurgery (15.7%), and neurology (15.2%). Anemia was more prevalent in the pulmonology unit (41.2%), the burns and reconstructive surgery unit (40.7%), and the thoracic surgery unit (38.5%). Clinical hospital unit types were significantly associated with anemia (*p* = 0.028). Anemia was significantly more frequent in patients with a medical history (38.2%) than in those without (23.6%). This relationship is statistically significant (*p* = 0.001) (Table 3). The classification of hospital diagnoses by clinical profile revealed a high frequency of anemia in neoplastic diseases (41.1%), followed by cardiorespiratory diseases (38.4%), infectious diseases (31.6%), and surgical diseases (27.7%). Analysis of the results showed a statistically significant association between anemia and the clinical classification of pathology groups (*p* = 0.019).

By comorbidity type, anemia was most frequent among patients with a history of hypertension (43.6%), diabetes (38.6%), dyslipidemia (38.9%), asthma (53.8%), heart disease (40.6%), and CKD (80%). A significant difference was found between anemia and hypertension (*p* = 0.000), diabetes (*p* = 0.03), and CKD (*p* = 0.03). No significant difference was observed with other comorbidities (Table 3).

#### 3.2.3. Distribution of Hematological Parameters

The median hemoglobin level (IQR) for study participants was 13.25 g/dL (12.07–14.5 g/dL), with a range of 2.9 to 18.8 g/dL. It was 12.80 g/dL (13.7–11.7) in women and 13.80 g/dL (15.1–12.4) in men. Median values for hematological parameters were 87.95 fL (IIQ: 57.4–105.6) for MCV, 33.20 pg (IIQ: 14–35.5) for TCMH, and 33.20 g/dL (IIQ: 23.6–37.8) for MCHC. The Median level was higher in men than in women for all three hematological parameters: Hb, MCV, and MCHC. We used the Mann–Whitney U test to compare Medians for Hb, MCV, and MCHC between the two sexes and according to anemia status (Table 4) The test reveals a statistically significant difference between the two sexes and between the anemic and non-anemic patient groups. Medians for hemoglobin, MCV, and MCHC were significantly lower in women than in men (*p*-value < 0.00). They were also lower in the anemic than in the non-anemic groups (*p*-value < 0.00).

#### 3.2.4. Classification of Anemias

**Figure 1:**
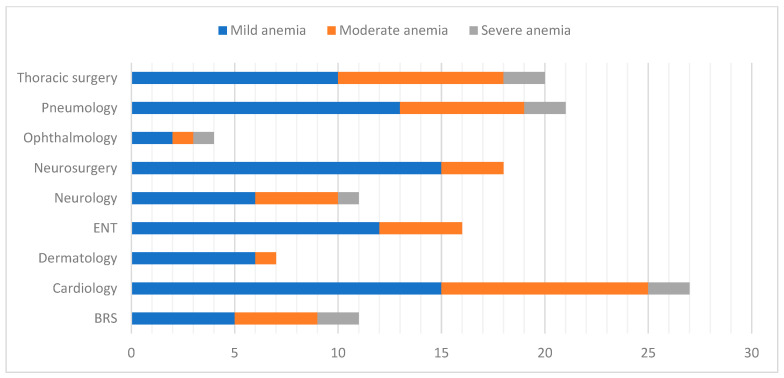
Anemia severity by hospital department among anemic patients (*n* = 135). Abbreviations: BRS: Burn and Reconstructive Surgery Unit, ENT: Otolaryngology.

According to Table 5, mild anemia (62.2%) is the predominant type in both sexes and across all hospital departments. Moderate anemia (30.4%) appeared as the second most frequent severity level, whereas severe anemia was only observed in a minority (7.4% of all anemia cases). Males predominated in the mild form of anemia (69.3%); however, no significant association between sex and anemia severity was observed. Fisher’s exact test showed a significant association between age class and anemia severity level (*p* = 0.022). Analysis of anemia severity by admission condition indicates that mild anemia was most prevalent across all clinical categories. Severe anemia occurred more frequently among patients admitted for infectious diseases. Patients hospitalized for inflammatory, surgical, and cardiorespiratory conditions primarily exhibited mild-to-moderate anemia. The distribution of anemia severity by hospital department among anemic patients confirms these findings. Mild anemia remains the predominant form in the majority of departments (Figure 1).

**Figure 2:**
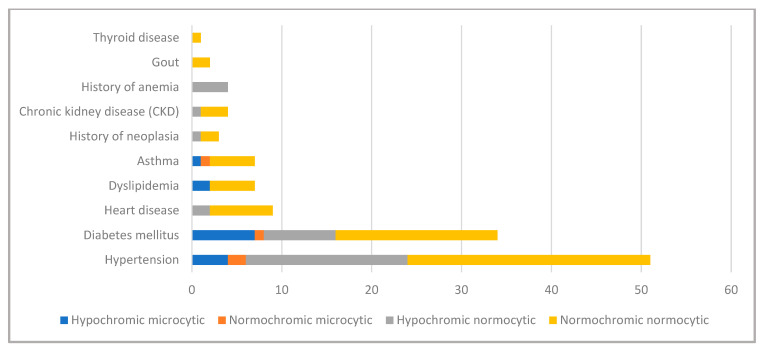
Distribution of anemia types according to medical comorbidities.

Regarding morphological patterns of anemia, normocytic normochromic anemia (56.3%) was the most common, followed by hypochromic normocytic anemia (24.4%), normochromic microcytic anemia (15.6%), and normochromic microcytic anemia (3.7%). This order was observed in the distribution of anemia morphological forms for both sexes (Table 6). The difference in anemia frequency by morphology and sex was statistically significant *(p*-value = 0.004). Analysis of the difference in morphological type of anemia by age group shows that the normocytic normochromic form is the most common in all age groups. This difference was not significant (*p* > 0.05). The analysis demonstrates that normochromic normocytic anemia was the predominant morphological type across most medical comorbidities, particularly among patients with hypertension, diabetes, and chronic kidney disease, while microcytic forms were less frequent (Figure 2). 

## 4. Discussion

The findings of this study provide updated evidence on the prevalence and characteristics of anemia among adults hospitalized in a tertiary care center in eastern Morocco. The overall prevalence of 30.3% observed at admission aligns with the range commonly reported in hospital-based studies worldwide. However, it remains slightly lower than the rates reported in units that manage complex chronic diseases or acute inflammatory conditions, where estimates often exceed 40% [11,30]. This difference is expected, given that our analysis excluded departments in which anemia is almost an inevitable comorbidity, such as internal medicine, nephrology, and intensive care. Nevertheless, the proportion remains clinically meaningful and highlights anemia as a frequent yet underrecognized condition in general hospital settings, underscoring the need for increased clinical awareness of anemia in routine hospital practice.

An important finding of this study is the marked increase in anemia with advancing age, particularly among individuals aged 60 years and older. This trend is consistent with international evidence indicating that aging is associated with a higher burden of comorbidities, chronic inflammation, renal impairment, and nutritional deficiencies, all of which contribute to impaired erythropoiesis [31,32]. The absence of a sex difference in our population contrasts with patterns observed in the general community, where women of reproductive age are more frequently affected [2], but is in line with several hospital-based studies showing that clinical comorbidities outweigh sex-related determinants once individuals reach older adulthood [33].

The association between anemia and comorbidities observed in this study is also well supported by the literature. Patients with hypertension, diabetes, or chronic kidney disease were significantly more likely to be anemic. These conditions promote anemia through diverse mechanisms, including impaired erythropoietin production, chronic low-grade inflammation, endothelial dysfunction, oxidative stress, and medication-related effects [34,35,36]. Such findings suggest that anemia may represent not only a hematologic disorder but also a surrogate marker of overall disease burden and physiological vulnerability. This perspective is increasingly emphasized in contemporary clinical research, where anemia has been linked to functional decline, reduced resilience to acute illness, and worse prognosis in multimorbid patients [21].

Morphologically, the predominance of normocytic normochromic anemia in our sample reinforces the likelihood that chronic disease mechanisms, rather than nutritional deficiencies, drive most cases identified at admission. The pathophysiology of anemia of inflammation, which involves impaired iron mobilization, increased hepcidin levels, shortened erythrocyte lifespan, and blunted erythropoietin response, explains many of the hematological patterns observed in our patients [8,37]. In contrast, microcytic hypochromic anemia, often indicative of iron deficiency, accounted for a smaller proportion of cases than typically observed in community-based Moroccan studies [24]. This discrepancy further distinguishes the hospital population from the general population and underscores the need to interpret anemia within its specific clinical context.

Normochromic normocytic anemia was the predominant morphological profile in patients with medical histories such as hypertension, diabetes, or chronic kidney disease. This profile is consistent with inflammatory anemia, frequently observed in the context of chronic infections, endocarditis, autoimmune inflammatory diseases, cancers, chronic kidney disease, heart failure, or inflammatory conditions associated with aging [38,39,40]. According to the literature, anemia of chronic diseases most often initially presents as normocytic and normochromic, then may progress to a microcytic and hypochromic form in cases of prolonged or severe inflammation [41,42]. Confirmation of this type of anemia requires a complete etiological workup [43].

Although most cases in this study were mild or moderate, their clinical implications remain significant. Even mild anemia can hinder postoperative recovery, increase infection risk, extend hospital stay, and raise the likelihood of readmission and mortality [12,20,22]. These consequences underscore the importance of adequate diagnostic workup and proactive management. However, as in other hospital-based studies, anemia is often underinvestigated, and a substantial proportion of patients are discharged without a clearly established etiology [44]. The present results highlight the opportunity to improve diagnostic pathways and to promote standardized assessment strategies as part of a broader Patient Blood Management approach [18].

The variation in anemia prevalence across hospital departments is another noteworthy observation. Higher rates were observed in pulmonary, thoracic surgery, and burn units, areas where chronic inflammation, repeated surgical interventions, persistent infections, and elevated metabolic demands are common. These clinical environments create physiological conditions conducive to anemia, underscoring the need for context-specific prevention and monitoring strategies. This finding also aligns with international trends, in which surgical and respiratory units frequently report preoperative or admission anemia as a significant predictor of adverse outcomes [19,33]. The distribution of patients according to hospitalization diagnosis confirms these results. The results of our study showed that the distribution of anemia varies by hospital pathology, with a high frequency in patients hospitalized for neoplastic, cardiorespiratory, and infectious conditions. These results have been well documented in the profile of inflammatory anemia. Several studies have reported that anemia is significantly associated with malignancy [33]. Anemia is very common in cancer patients, and its mechanism is linked to both the disease and its treatments [45], with a prevalence of 50.3% among patients with malignant tumors [46] and 41.5% in women with cancer [47]. Anemia is a common comorbidity in hospitalized patients, and the importance of underlying diseases is supported by the literature. Anemia is a common comorbidity in cardiac diseases [48] and identifies a high prevalence of anemia among patients hospitalized for heart failure, with rates ranging from 33% to 70% [49,50]. Furthermore, anemia has been observed in 23.5% of patients with chronic obstructive pulmonary disease [51] and among 50% of people living with HIV [52]. Generally, the clinical profile of anemias observed in this study aligns with previous research, which indicates that anemia is frequently associated with chronic inflammatory diseases and a high burden of comorbidities [11,53,54].

In this study, regardless of patient age, anemia was associated with hospitalization-specific clinical factors. This finding underscores the need for systematic assessment and proactive management of anemia in hospital settings, particularly as anemia related to the cause of hospitalization significantly impacts hospital mortality and morbidity. The challenge of managing anemia during hospitalization can be complicated by the development of hospital-acquired anemia, which has been shown to have a high frequency and negative clinical outcomes [55,56].

Anemia is an indicator of health status, whose diagnosis and treatment can improve patients’ physical and functional capacities [32]. Despite the serious consequences of anemia for the health and prognosis of hospitalized patients, a review of the literature indicates that anemia management is often neglected in patients hospitalized for reasons other than anemia. Moreover, in a study conducted in the United Kingdom, more than half of anemic patients received no management or follow-up on discharge [57]. The underdiagnosis of anemia in the hospital setting has been confirmed by a recent study conducted at the University of Chicago Medical Center (UCMC) among 945 anemic patients hospitalized with Hb < 10 g/dL. The study showed that etiological investigations of anemia are not systematically performed in hospitalized patients, and 37% of cases remain without an identified cause [44].

The anemia management circuit in Morocco is based on a care pathway that begins with primary healthcare at the health center level, which serves as the first point of contact for consultations outside emergencies, and extends to the tertiary level. This university hospital covers one or more regions. As with any pathology, screening should be carried out by the general practitioner at the health center, who will either monitor the patient or refer them to a more specialized structure for appropriate treatment. Since the hospital environment offers the opportunity to screen for anemia (through systematic entry assessment in most units), anemia must be screened for, complementary examinations initiated, and the patient referred for anemia management upon discharge.

In Morocco, most previous research on anemia has focused on children and women of childbearing age. National data on anemia are based on the latest national survey (2019–2020), which studied the prevalence of anemia in women of childbearing age and children. In hospital settings, most studies have focused on anemic patients. Thus, in our context, studies on the epidemiological profile of anemia in adults hospitalized for conditions other than anemia remain scarce, and to date, no study has offered detailed profiling across multiple hospital departments. In this context, our study complements previous anemia research by adopting a cross-sectional approach.

Anemia is a multifactorial condition, considered both a pathology and a symptom of underlying, sometimes serious, illnesses. Given limited resources and its often-silent nature, the hospital provides a pragmatic setting for anemia screening, given the availability of complete blood counts (CBCs). The benefit of this approach is therefore twofold: clinical, by promoting early diagnosis and better consideration of severity, and practical, by contributing to the improvement of screening and management of anemia in routine hospital settings.

This study, conducted in a leading university hospital in eastern Morocco, aims to fill this gap by providing relevant data adapted to the local context. These findings can inform hospital policies, guide clinical decision-making, and support national efforts to improve the surveillance and management of anemia. The study also highlights the need for better coordination between hospital departments and general practitioners to ensure continuity of follow-up and treatment after hospital discharge, particularly for patients with chronic diseases.

A limitation of our study is its cross-sectional design. This collects exposure and outcome at a single point in time. As a result, it limits the determination of temporality and causal relationships. The study also uses convenience sampling and was conducted in a single hospital. This monocentric approach may limit generalizability to other contexts or the Moroccan population. Future multicenter research is needed to validate these results in different populations. Prospective longitudinal studies will also clarify the association between anemia and associated factors. However, our study demonstrates several important methodological advantages. To the best of the authors’ knowledge, this study is the first to report anemia prevalence among admitted patients across multiple hospital departments in Morocco. Its relevance is twofold. First, it offers an original perspective by examining anemia in adults, a population that is rarely studied. Second, it was conducted at a tertiary referral hospital serving the entire Eastern Moroccan region. The findings have important epidemiological and clinical implications. They provide healthcare teams with evidence of the high prevalence of anemia at admission and, from an epidemiological standpoint, offer data to strengthen anemia management in hospitals and the broader health system.

## 5. Conclusions

This study demonstrates that anemia is a common finding among adults admitted to a tertiary hospital in eastern Morocco, with a prevalence of 30.3% and a predominance of normocytic normochromic profiles, suggesting underlying chronic disease mechanisms. The strong associations observed with advanced age, hypertension, diabetes, and chronic kidney disease highlight the interplay between anemia and multimorbidity. Although most cases were mild or moderate, their potential clinical consequences justify systematic screening at admission and prompt etiological assessment. Strengthening diagnostic pathways, promoting Patient Blood Management principles, and improving coordination between inpatient and outpatient care could collectively enhance the management of anemia in Morocco. Future multicenter, longitudinal, population-based studies are warranted to evaluate long-term outcomes associated with anemia and to identify strategies to reduce its burden in hospitalized populations.

## Figures and Tables

**Table 1:** Socio-demographic, economic, and lifestyle characteristics of study participants according to anemia status (*N* = 446).

Variable	Category	Total Patients *n* (%)	Non-Anemic *n* (%)	Anemic *n* (%)	*p*-Value
	Total	446 (100%)	311 (69.7)	135 (30.3)	
Age (years)	median (IQR)	57 (41.75–68)	56 (40–66)	62 (47–71)	
Age range	18–29 years	52 (11.7)	36 (69.2)	16 (30.8)	0.004 ^a^
	30–44 years	78 (17.5)	64 (82.1)	14 (17.9)	
	45–59 years	114 (25.6)	86 (75.4)	28 (24.6)	
	60 years and over	202 (45.3)	125 (61.9)	77 (38.1)	
Sex	Men	235 (52.7)	160 (68.1)	75 (31.9)	0.425 ^a^
	Women	211 (47.3)	151 (71.6)	60 (28.4)	
Marital status	Single	85 (19.1)	64 (75.3)	21 (24.7)	0.289 ^a^
	Divorced	20 (4.5)	11 (55)	9 (45)	
	Married	303 (67.9)	208 (68.6)	95 (31.4)	
	Widowed	38 (8.5)	28 (73.7)	10 (26.3)	
Place of residence	Rural	139 (31.2)	212 (69.1)	95 (30.9)	0.644 ^a^
	urban	307 (68.8)	99 (71.2)	40 (28.8)	
Provenance	Berkane	68 (15.2)	52 (76.5)	16 (23.5)	0.819 ^b^
	Driouch	9 (2)	5 (55.6)	4 (44.4)	
	Figuig	13 (2.9)	8 (61.5)	5 (38.5)	
	Guercif	31 (7)	22 (71)	9 (29)	
	Jerada	24 (5.4)	17 (70.8)	7 (29.2)	
	Nador	59 (13.2)	38 (64.4)	21 (35.6)	
	Oujda	195 (43.7)	137 (70.3)	58 (29.7)	
	Taourirt	47 (10.5)	32 (68.1)	15 (31.9)	
Education level	Illiterate	222 (49.8)	151 (68)	71 (32)	0.364 ^a^
	No formal	15 (3.4)	10 (66.7)	5 (33.3)	
	Primary	88 (19.7)	57 (64.8)	31 (35.2)	
	Secondary	94 (21.1)	72 (76.6)	22 (23.4)	
	Higher or above	27 (6.1)	21 (77.8)	6 (22.2)	
Occupational status	Formal	90 (20.2)	68 (15.2)	22 (4.9)	0.594 ^b^
	Informal	5 (1.1)	4 (0.9)	1 (0.2)	
	Unemployed	313 (70.2)	213 (47.8)	100 (22.4)	
	Inactive	38 (8.5)	26 (5.8)	12 (2.7)	
Health insurance coverage	AMO TADAMON	233 (52.2)	165 (70.8)	68 (29.2)	0.591 ^a^
	Other insurances	10 (2.2)	9 (90)	1 (10)	
	CNOPS	37 (8.3)	26 (70.3)	11 (29.7)	
	CNSS	81 (18.2)	55 (67.9)	26 (32.1)	
	Uninsured	85 (19.1)	56 (65.9)	29 (34.1)	
Physical activity	No	371 (83.2)	254 (68.5)	117 (31.5)	0.122 ^a^
	Yes	75 (16.8)	57 (76)	18 (24)	
Smoking status	Current smoker	33 (7.4)	200 (69)	90 (31)	0.873 ^a^
	Former smoker	123 (27.6)	88 (71.5)	35 (28.5)	
	Non-smoker	290 (65)	200 (69)	90 (31)	
Alcohol consumption	Yes	17 (3.8)	10 (58.8)	7 (41.2)	0.318 ^a^
	No	429 (96.2)	301 (70.2)	128 (29.8)	

^a^ Pearson’s Chi-square test; ^b^ Fisher’s exact test. AMO TADAMON: Moroccan public health insurance scheme for low-income populations. CNOPS: National Fund for Social Welfare Organizations; CNSS: National Social Security Fund.

**Table 2:** Distribution of anemia prevalence (and association with age) in both sexes, by age group.

Age Group (Years)	Total *n* (%)	Non-Anemic *n* (%)	Anemic *n* (%)	*p*-Value
Men (N = 235)				0.007
18–29	31 (13.2%)	20 (64.5%)	11 (35.5%)	
30–44	31 (13.2%)	28 (90.3%)	3 (9.7%)	
45–59	59 (25.1%)	44 (18.7%)	15 (6.4%)	
60 and over	114 (48.5%)	68 (59.6%)	46 (40.4%)	
Women (N = 211)				
18–29	21 (10%)	16 (76.2%	5 (23.8%)	0.331
30–44	47 (22.3%)	36 (76.6%)	11 (23.4)	
45–59	55 (26.1%)	42 (76.4%)	13 (23.6%)	
60 and over	88 (41.7%)	57 (64.8%)	31 (35.2%)	

*p*-values were calculated using Pearson’s Chi-square test.

**Table 3:** Association between anemia status and clinical hospitalization parameters in the study population (N = 446).

Variable	Category	Total Patients	Anemia Status		
		Frequency (%)	Non Anemic *n* (%)	Anemic *n* (%)	*p*-Value
	Total patients	(*n* = 446)	311 (69.7)	135 (30.3)	
Hospitalization unit	BRS	27 (6.1)	16 (59.3)	11 (40.7)	**0.028**
	Cardiology	73 (16.4)	46 (63)	27 (37)	
	Thoracic surgery	52 (11.7)	32 (61.5)	20 (38.5)	
	Dermatology	32 (7.2)	25 (78.1)	7 (21.9)	
	Neurology	68 (15.2)	57 (83.8)	11 (16.2)	
	Neurosurgery	70 (15.7)	52 (74.3)	18 (25.7)	
	Ophthalmology	22 (4.9)	18 (81.8)	4 (18.2)	
	ENT	51 (11.4)	35 (68.6)	16 (31.4)	
	Pneumology	51 (11.4)	30 (58.8)	21 (41.2)	
Clinical hospital units	Medical	158 (70.5%)	66 (29.5%)	66 (48.9)	0.658
	Surgical	100 (67.1%)	49 (32.9%)	49 (36.3)	
	Medical–Surgical	53 (72.6%)	20 (27.4%)	20 (14.8)	
Admission diagnosis category (*n* = 426)	Cardio-respiratory	112 (26.3)	69 (61.6%)	43 (38.4%)	0.019
	Surgical	94 (22.1)	68 (72.3%)	26 (27.7%)	
	Infectious	38 (8.9)	26 (68.4%)	12 (31.6%)	
	Inflammatory	85 (20)	69 (81.2%)	16 (18.8%)	
	Neoplastic	56 (13.1)	33 (58.9%)	23 (41.1%)	
	Others	41 (9.6)	32 (78%)	9 (22%)	
The prevalence of different comorbidities					
Medical history	No	242 (54.3)	185 (76.4)	57 (23.6)	**0.001**
	Yes	204 (45.7)	126 (61.8)	78 (38.2)	
Hypertension	No	329 (73.8)	245 (74.5)	84 (25.5)	**<0.001**
	Yes	117 (26.2)	66 (56.4)	51 (43.6)	
Diabetes	No	358 (80.3)	257 (71.8)	101 (28.2)	**0.039**
	Yes	88 (19.7)	54 (61.4)	34 (38.6)	
Dyslipidemia	No	428 (96)	300 (70.1)	128 (29.9)	0.416
	Yes	18 (4)	11 (61.1)	7 (38.9)	
Asthma	No	433 (97.1)	305 (70.4)	128 (29.6)	0.071 ^b^
	Yes	13 (2.9)	6 (46.2)	7 (53.8)	
Heart disease	No	424 (95.1)	298 (70.3)	126 (29.7)	0.340
	Yes	22 (4.9)	13 (59.1)	9 (40.9)	
Chronic Kidney Disease	No	441 (98.9)	310 (70.3)	131 (29.7)	**0.031 ^b^**
	Yes	5 (1.1)	1 (20)	4 (80)	

*p*-values were calculated using Pearson’s chi-square test; *p*-values marked with superscript (b) were obtained using Fisher’s exact test. BRS: Burn and Reconstructive Surgery; ENT: Ear, Nose and Throat. Bold indicates statistical significance (*p* < 0.05).

**Table 4:** Hematological parameters of the study population by anemia status (N = 446).

Hematological Parameters	Total (*n* = 446) Median (IQR)	Anemic (*n* = 235) Median (IQR)	Not Anemic (*n* = 211) Median (IQR)	*p*-Value *
Hemoglobin (g/dL) (2.9–18.8) Masculin (6.1–16.6) Féminin	13.25 (12.07–14.5)	11.4 (10.3–12)	13.9 (13.2–14.9)	<0.001
MCV (57.4–105.6) Masculin (60.4–100.5) Féminin	87.95 (84.6–91.5)	85.90 (81.6–89.5)	88.7 (85.7–91.8)	<0.001
MCHC (23.6–37.8) Masculin (25.6–36.8) Féminin	33.20 (32–34)	32.4 (31.3–33.3)	33.4 (32.5–34.4)	<0.001
MCH (14–35.5) Masculin (18–33) Féminin	29.4 (28–30.6)	29.8 (28.6–30.9)	29.8 (28.6–30.9)	<0.001

* *p* < 0.05 of the Mann–Whitney U test was considered significant. MCV: Mean Corpuscular Volume; MCHC: Mean Corpuscular Hemoglobin Concentration; MCH: Mean Corpuscular Hemoglobin.

**Table 5:** Association between anemia severity and age, sex, and admission diagnosis in anemic patients (*n* = 135).

		Mild Anemia	Moderate Anemia	Severe Anemia	*p*-Value
Age range	18–29	10 (62.4%)	3 (18.8%)	3 (18.8%)	0.022 ^b^
	30–44	9 (64.3%)	4 (28.6%)	1 (7.1%)	
	45–59	13 (46.4%)	10 (35.7%)	5 (17.9%)	
	60 and over	52 (67.5%)	24 (31.2%)	1 (1.3%)	
Sex	Male	52 (69.3%)	19 (25.3%)	4 (5.3%)	0.152 ^a^
	Female	32 (53.3%)	22 (36.7%)	6 (10%)	
Total	Total (135)	84 (62.2%)	41 (30.4%)	10 (7.4%)	
Admission Diagnosis (*n* = 129)	Cardio-respiratory	24 (55.8%)	16 (37.2%)	3 (7.0%)	0.846 ^b^
	Surgical	18 (69.2%)	7 (26.9%)	1 (3.8%)	
	Infectious	7 (58.3%)	3 (25.0%)	2 (16.7%)	
	Inflammatory	13 (81.3%)	2 (12.5%)	1 (6.3%)	
	Neoplastic	16 (69.6%)	7 (30.4%)	-	
	Others	4 (44.4%)	4 (44.4%)	1 (11.1%)	

^a^: *p*-values computed using Pearson’s chi-square test; ^b^: *p*-values calculated using Fisher’s exact test.

**Table 6:** The frequency and association of morphological patterns of anemia among anemic participants with sex and age groups.

		Normocytic Normochromic *n* (%)	Normocytic Hypochromic *n* (%)	Microcytic Hypochromic *n* (%)	Normochromic Microcytic *n* (%)	*p*-Value ^a^
Total	*n* = 135	76 (56.3)	33 (24.4)	21 (15.6)	5 (3.7)	
Sex	Female	25 (41.7)	21 (35)	13 (21.7)	1 (1.7)	0.004
	Male	51 (68)	12 (16)	8 (10.7)	4 (5.3)	
Age range (years)	18–29	9 (56.3)	1 (6.3)	6 (37.5)	0	0.121
	30–44	8 (57.1)	4 (28.6)	2 (14.3)	0	
	45–59	17 (60.7)	4 (14.3)	6 (21.4)	1 (3.6)	
	60 and over	42 (54.5)	24 (31.2)	7 (9.1)	4 (5.2)	

^a^: *p*-values calculated using Fisher’s exact test.

## Data Availability

The datasets generated and analyzed for the current study are available from the corresponding author upon reasonable request, due to confidentiality restrictions.

## Abbreviations

The following abbreviations are used in this manuscript:

BRS	Burn and Reconstructive Surgery Unit
CBC	Complete Blood Count
CKD	Chronic Kidney Disease
CNOPS	National Fund for Social Welfare Organizations
CNSS	National Social Security Fund
ENT	Otolaryngology
HGB	Hemoglobin
IQR	Interquartile Range
MCH	Mean Corpuscular Hemoglobin
MCHC	Mean Cell Hemoglobin Concentation
MCV	Mean Corpuscular Volume
WHO	World Health Organization
